# Bioavailability of calcium in an enriched postbiotic system compared to calcium citrate in healthy postmenopausal females; A randomized, double-blind, comparator-controlled, crossover study

**DOI:** 10.3389/fnut.2023.1073622

**Published:** 2023-03-15

**Authors:** Marina Friling, Adi Haber, Sharon Furman-Assaf, David Israel, Gil Harari, Malkanthi Evans, David C. Crowley, Arthur C. Ouwehand, Eran Ivanir

**Affiliations:** ^1^IFF Health, Migdal Haemeq, Israel; ^2^Medistat, Tel-Aviv, Israel; ^3^KGK Science Inc., London, ON, Canada; ^4^IFF Health, Kantvik, Finland

**Keywords:** bioavailability, absorption, calcium supplements, calcium citrate, postbiotics, yeast, *Lactobacillus*, post menopause

## Abstract

**Introduction:**

Bioavailability of calcium is an important consideration when designing supplements for achieving adequate calcium intake, mainly in high-risk, and aged populations. Alternative supplementation strategies may be able to circumvent absorption issues commonly seen with calcium supplements. The objective of this study was to assess the bioavailability of a single serving of two calcium formulations vs. comparator product in healthy postmenopausal women.

**Methods:**

A total of 24 participants between 45 and 65 years were enrolled in a randomized, double-blind, three-phase, crossover study, with a 7-day washout period between phases. The bioavailability of calcium from calcium-carrying *Saccharomyces cerevisiae* (Ca-SC) or calcium-carrying *Lactobacillus* (Ca-LAB) in the form of postbiotic products versus calcium citrate, a conventional salt-based calcium supplement, was determined. Each product provided 630 mg of calcium and 400 IU of vitamin D3. After a 14-h (overnight) fast followed by a single dose of product with a standard low-calcium breakfast, both serum and urine calcium concentrations were assessed for up to 8 and 24 h, respectively.

**Results:**

Ca-LAB resulted in greater calcium bioavailability, demonstrated by significantly higher area under the curve and peak concentration both in blood and urine, and total calcium mass excreted in urine. The bioavailability of calcium was similar for Ca-SC and calcium citrate except for the peak concentration value that was significantly higher for calcium citrate. Both Ca-LAB and Ca-SC were well tolerated with no significant difference in adverse events between the products during the study.

**Discussion:**

These findings suggest that calcium enriched in a *Lactobacillus*-based postbiotic system is associated with higher levels of bioavailability as compared to calcium citrate, while a calcium-enriched yeast-based postbiotic does not influence calcium absorption.

## 1. Introduction

Calcium is an essential mineral for the proper function of the body. The structural role of calcium in the body is well-documented, as well as its role in a wide range of biologic functions such as bone formation, muscle contraction, extra-and intracellular signaling and nerve impulse transmission (1, 2). Maintaining calcium homeostasis is crucial for human health, and it is largely regulated by vitamin D and its derivatives (3, 4). Short-term deficiencies in calcium are asymptomatic since blood calcium is held in tight homeostasis. Chronic deficiencies are associated with numb and tingling fingers, cramping, lethargy, abnormal heart rhythm, and bone diseases such as osteomalacia and osteoporosis (5).

Osteoporosis and other bone disorders are growing burdens on national healthcare systems as they can lead to fracture injuries, which require resource-intensive treatments. Especially older individuals are more susceptible to bone loss and associated complications due to age-related changes in sex steroid production, muscle mass, and muscle quality (6–8). Also, post-menopausal women are at increased risk of losing bone mass and developing osteoporosis because of the marked decrease in estrogen associated with menopause (9–11). Estrogen is an anti-resorptive agent, so the decrease in estrogen leads to an increase in resorption of calcium from bones and a decrease in calcium absorption from the diet (3, 10, 12). Osteoporosis-related bone loss is most dramatic in the first few years after menopause, when women have been reported to lose 3–7% of total bone mass per year. This figure typically drops to under 2% per year by age 65 (13).

Calcium is only available to the body through exogenous sources, primarily *via* the diet. The main dietary sources of calcium are dairy products and certain vegetables although also calcium fortified plant-based ‘milks’ and calcium rich mineral waters can be sources of dietary calcium. Calcium supplementation is usually indicated when dietary calcium intake may be insufficient (14). According to the National Institutes of Health (NIH), roughly 43% of all Americans and 70% of elderly American women use calcium supplements (15). The NIH recommends a calcium intake of 1,000 mg/day for adults to promote bone health. Recommendations increase to 1,200 mg/day for older adults and postmenopausal women who are at higher risk of developing bone disease such as osteoporosis. Currently, salt-based supplements including calcium carbonate and calcium citrate are popular because they are inexpensive (5). Although dietary calcium absorption varies and is dependent on the food matrix, calcium supplements may not be absorbed as effectively as intended (16, 17), and may contribute to kidney stone formation (18, 19) and gastrointestinal symptoms including constipation and bloating (14, 20).

Bioavailability of calcium is an important consideration when designing supplements because high intake is meaningless if absorption is limited. This is of interest in high-risk, aged populations, since they have been shown to have a decreased ability to absorb calcium (21, 22). Vitamin D and its derivatives are important regulators of serum and bone calcium levels, so vitamin D deficiency can negatively impact calcium uptake (3). Calcium from dairy products has been documented to be absorbed efficiently. Cooked dark green leafy vegetables and *Brassica* sp. vegetables have been shown to be important dietary sources of calcium, although calcium absorption can be affected by the presence of oxalate and phytate (23). Variability in absorption between supplemental and dietary intake has been attributed to several factors such as interactions of calcium with other food components in the food matrix and its uptake at targeted sites throughout the gastrointestinal tract (16, 17, 24). Alternative supplementation strategies may circumvent absorption issues reported with calcium salt supplements (25–28). Calcium from calcium-loaded *Saccharomyces cerevisiae* (Ca-SC) in the form of a postbiotic system has been investigated as a potential alternative to traditional supplements. Since calcium is integrated into the structure of the yeast, it is hypothesized that a supplement made from calcium-enriched yeast would behave similarly to food in the gastrointestinal system and, as such, have greater uptake than traditional supplements. This theory has been supported by a urine excretion study for Ca-SC (29) as well as in other enriched yeast systems (30–32). We also hypothesized that calcium-loaded *Lactobacillus* (Ca-LAB) postbiotic is better absorbed than calcium salts for a similar reason.

The current study compares calcium bioavailability of postbiotics Ca-LAB, Ca-SC to calcium citrate using classic pharmacokinetic parameters based on changes in serum and urine calcium concentrations. The postmenopausal women population were selected due to their higher risk of losing bone mass and developing osteoporosis from the marked decrease in estrogen associated with menopause (9, 11).

## 2. Methods

### 2.1. Study design

**Figure 1:**
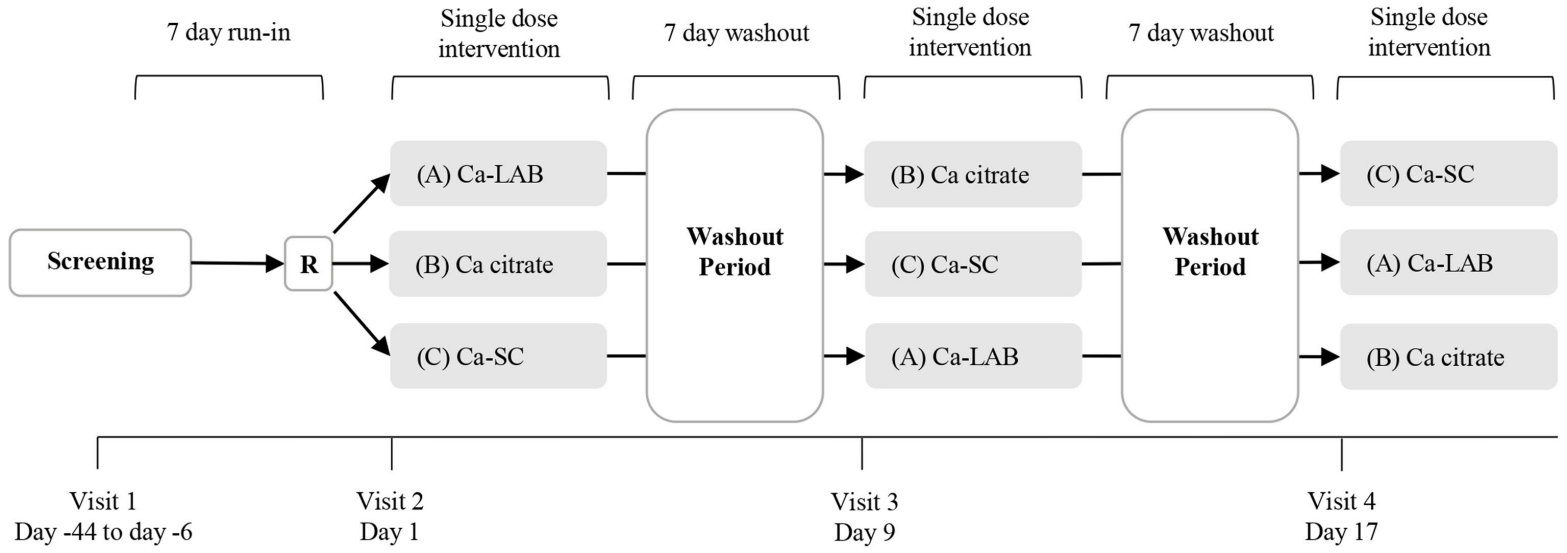
Overview of study design. This was a double-blind, randomized, three-phase, crossover study. Intervention phases were separated by a 7-day washout period. Participants were randomly assigned into one of three intervention sequence arms at visit 2. R = randomization; Ca = calcium.

This was a double-blind, randomized, comparator controlled, three-phase, cross-over clinical trial conducted at KGK Science (London, ON, Canada) between 18th December 2019 and 26th January 2021. The study compared the bioavailability of three calcium products (Ca-SC, Ca-LAB, and calcium citrate) in healthy postmenopausal women. Each participant took part in three single dose administrations with two washout periods of 7 days between interventions, as shown in Figure 1. The study protocol was reviewed by Advarra Institutional review board (Aurora, ON, Canada) and Health Canada’s Natural and Non-prescription Health Products Directorate, and an unconditional approval was received. IRB protocol # Pro00040124, Health Canada Notice of Authorization # 246340. The study was conducted in compliance with the international council for harmonization of technical requirements for pharmaceuticals for human use (ICH) Guideline for good clinical practice (GCP) and in accordance with the Declaration of Helsinki guidelines and its subsequent amendments and followed the CONSORT guidelines for randomized controlled trials (33, 34). All participants provided written informed consent prior to any study procedures and were told that they could withdraw from the study at any time (35).

### 2.2. Participants

Participants were included if: 45–65 years of age with a body mass index (BMI) between 19.0 to 29.9 kg/m^2^; postmenopausal as defined by no menstrual period *via* natural processes for at least 12 months; had normal electrocardiogram (ECG); parathyroid hormone (1–84 PTH) between 1.6 and 6.9 pmol/L; 25-hydroxyvitamin D [25(OH)D] level ≥ 20 ng/mL; and had a calcium intake of >450 mg per day from diet and supplementation (36–39). Participants were excluded if they had: an allergy, sensitivity, or intolerance to the investigational product’s active, inactive ingredients or the ingredients of the standardized meals; any disease that affected Calcium or vitamin D metabolism; or had any other condition that in the medical director’s opinion would affect the participants ability to complete the study or its measures or pose a significant risk to the participant. Detailed inclusion and exclusion criteria are specified in Supplementary Tables S1, S2.

### 2.3. Investigational products and dosage

Each participant completed three intervention visits, consuming a single serving of two calcium formulations and a reference product. Ca-SC (Re-Natured® calcium) and Ca-LAB (Biogurt™ calcium) are both derived from enriched calcium cultures of *S. cerevisiae* and *Lactobacillus delbrueckii* subsp. *bulgaricus-87*, respectively, (Grow Company, Ridgefield, NJ, USA) (40). The cells were grown in the presence of calcium, heat-killed at the end of production process and spray dried into a dried powder, delivering inactive cells. Subsequently, vitamin D3 (Xiamen Kingdomway group Company, Xiamen, China) was added to the dried powders, and the mixed formulations were encapsulated in identical capsules, providing each 630 mg calcium and 400 IU vitamin D3 per serving. The reference product was calcium citrate tablets (Bayer, Leverkusen, and Germany), containing 315 mg calcium and 200 IU vitamin D3 per tablet. The tablets were grinded into a powder form and encapsulated in the same capsule format as the experimental products, providing 630 mg Calcium and 400 IU vitamin D3 per serving. Encapsulation of the powders was conducted under good manufacturing practice (GMP) by an independent company. After encapsulation, the three products were analyzed to assure standardized and identical quantities per serving. Calcium content was analyzed by inductively coupled plasma-optical emission spectrometry (ICP-OES), and vitamin D content was analyzed by high performance liquid chromatography (HPLC). All products were stored at room temperature, protected from moisture and direct light until administration.

### 2.4. Randomization

Participants who were found eligible were assigned a randomization code on day 1 and randomly allocated into one of three intervention sequence arms: A➔B➔C, B➔C➔A, or C➔A➔B according to the randomization list: (A) A Single dose of Ca-LAB (investigational product), (B) A Single dose of calcium citrate (reference product), or (C) A single dose of Ca-SC (investigational product). Each arm comprised eight participants. The randomization schedule was generated using www.randomization.com.

### 2.5. Blinding

Study products were labeled with an individual randomization and visit number and were identical in appearance, texture, and taste. Labeling was done by designated personnel not involved in other aspects of the study to maintain blinding. Intervention allocation was concealed by using opaque sealed envelopes, each labelled with a randomization number. Each envelope contained information regarding the intervention associated with each randomization number. Investigators and study staff remained blinded to the assigned sequence until the database was locked, and the data were analyzed.

### 2.6. Study protocol

Participants underwent screening procedures within 37 days prior to the study run-in period (7 days) to assess their eligibility for participation. At the screening, written consent was obtained from each participant prior to the conduct of any study-related activity. Once consent was obtained, medical history, concomitant medications and current health status were reviewed, seated systolic and diastolic blood pressure (SBP and DBP), heart rate (HR), oral temperature, height, weight, and BMI were measured, and an ECG was recorded. In addition, fasted blood and urine samples were collected for analysis. Blood test included: complete blood count (CBC), electrolytes (sodium, potassium, chloride, phosphate), total calcium, hemoglobin A1c (HbA1c), fasting glucose, thyroid stimulating hormone (TSH), estimated glomerular filtration rate (eGFR), creatinine, alanine aminotransferase (ALT) and aspartate aminotransferase (AST), alkaline phosphatase (ALP), albumin, total bilirubin, 1–84 PTH, 25(OH)D, low-density lipoprotein cholesterol (LDL-C), and triglycerides. Urine test included: total calcium, creatinine, and albumin. Nutritional counselling and nutritional guidelines were provided. From 1 week prior to baseline (day 1) to the end of the study, participants were instructed to restrict their calcium and sodium dietary intake to 400 and 2,300 mg/day, respectively. These dietary instructions were intended to provide a high level of standardization in terms of the level of calcium concentration/metabolism (39). Prior to the intervention days, i.e., day’s −1, 8, and 16, participants fasted for 14 h and were only instructed to drink 600 mL distilled water 12 h prior to administration and 300 mL distilled water 9 h prior to administration. These activities were recorded in the participants’ study diary.

On the intervention days, i.e., days 1, 9, and 17, participants were instructed to empty their bladder (including the first urine of the morning) 2 h prior to administration time and consume 600 mL distilled water. During this time, participants also began collecting all their pre-intervention urinary output. Once attending the study site, participants were under continuous medical supervision until 10 h post dose. At the study site, seven-day food record was reviewed and concomitant therapies, health status and vitals (BP, HR, and oral temperature) were assessed. Under fasting conditions, a pre-intervention blood sample was collected, and participants emptied their bladder again and the overall urinary output was collected over a time window of 2 h prior administration. Then, participants consumed a standardized low-calcium breakfast (scrambled egg made of three egg whites cooked with one teaspoon olive oil, one slice sourdough bread with one teaspoon butter, and one cup of raw watermelon; overall calcium content of 39.2 mg) with 600 mL distilled water and half-way through consumed the investigational product. Participants drank 300 mL of distilled water every 2 h post dose and were given a standard lunch low-calcium meal (90 grams roasted beef, 80 grams banana, and three cups of salad containing: iceberg lettuce, tomato, cucumber, and onion with one tablespoon vinaigrette; overall calcium content of 50 mg) 6 h after the standard breakfast. The main purpose for the standardized low-calcium was to reduce the risk of confounding outcomes. Vital signs (BP, HR, and oral temperature) were collected every 2 h up to 10 h post-dose. Blood samples were collected at 1, 1.5, 2, 2.5, 3, 3.5, 4, 5, 6, 7 and 8 h. Urine samples were collected for time frames 0–2, 2–4, 4–6, 6–8, 8–10 and 10–24 h. At 10 h post dose, participants drank 600 mL of distilled water at home and returned to the study site the next morning to submit their 10–24 h urine container. Before each intervention day, there was a 7 ± 2 days washout period where participants completed a seven-day food record to monitor compliance with the restricted calcium and sodium dietary intake between visits. The completed food records were reviewed by clinic staff prior to starting assessments on each intervention day to ensure participants complied with the dietary instructions.

### 2.7. Sample preparation and analysis

#### 2.7.1. Blood calcium

Blood was collected in 5 mL serum separator tubes (SST) clot activator with gel (BD Vacutainer, Mississauga, ON, Canada) for serum calcium analysis. Serum collected from pre-dose and each post-dose time interval was stored at 4°C until the end of each collection period and sent for analysis. Serum calcium was analyzed using the Roche c701/c501 equipment by colorimetric assay, at Dynacare Laboratory (Brampton, ON, Canada).

#### 2.7.2. Urine calcium and creatinine

Urine collected from pre-dose and each post-dose time interval was stored at 4°C until the end of each collection period. At the end of each collection period, total urine volumes collected at each post-dose time interval were measured, and the urine container agitated to ensure homogeneity. A sample was aliquoted for urine creatinine which was used to normalize calcium in the urine. For urine calcium, 20 mL of 6 M hydrochloric acid was added per 1,000 mL urine and centrifuged at 1,000 RCF for 20 min at 4°C. An aliquot of the acidified urine was stored at 4°C until the end of each collection period and sent for analysis. Urine calcium and creatinine were analyzed using the Roche c701/c501 equipment by colorimetric and enzymatic methods respectively, at Dynacare Laboratory (Brampton, ON, Canada).

### 2.8. Assessments

#### 2.8.1. Bioavailability parameters

Incremental area under the curve (iAUC) from 0 to 8 h was calculated for serum calcium by determining the area under the curve above the baseline value using the trapezoid method. Total area under the curve (AUC_T_) from 0 to 8 h for serum calcium, and 0 to 10 and 24 h for urine calcium were derived from individual concentration-time plots for each participant using the trapezoid method. Urinary calcium was corrected by corresponding urinary creatinine to accommodate for the completeness of the urine collection. Peak concentration of serum and urine calcium (*C*_max_) and time to peak concentration for serum calcium (*t*_max_) were determined directly from the concentration-time curve. For urine, *t*_max_ was calculated as the median of the collection interval at the observed peak concentration. In addition, cumulative urinary calcium excretion after 10 and 24 h were calculated.

#### 2.8.2. Safety evaluation

All participants who received at least one dose of any study product were included in the safety evaluations. Safety analyses include vital signs (blood pressure, heart rate, oral temperature), anthropometrics (height, weight, and BMI), biochemistry analysis (ALT, AST, albumin, creatinine, and electrolytes (sodium, potassium, and chloride), fasting and random glucose, and total bilirubin), hematology analysis (white blood cell (WBC) differential count, red blood cell (RBC) count, hemoglobin, hematocrit, platelet count, and RBC indices), and incidence of adverse events (AEs). All clinical laboratory analysis were conducted using standardized procedures at Dynacare Laboratory (Brampton, ON, Canada).

Adverse events were recorded throughout the study using a study diary and were reviewed at each study visit to classify based on the description, duration, intensity, frequency, and outcome. All AEs were analyzed by Medical Dictionary for Regulatory Activities (MedDRA) dictionary Version 24.0. Severity and causal relationship with the product were assessed by the qualified investigator (QI).

### 2.9. Statistical analyses

A sample size of 20 participants, with a total of 24 assuming 20% attrition rate, was estimated to detect a difference in pharmacokinetic parameters with an effect size of *d* = 0.66 and 80% power using a paired *t*-test with 0.05 two-sided significance level (41). Head-to-head comparisons between the calcium products were made with respect to pharmacokinetic parameters based on the intent-to-treat (ITT) population, using a mixed model Analysis of Variance (ANOVA), with product, sequence and period as fixed effects, and participant as the random effect. Pharmacokinetic parameters that were not distributed normally by the Wilk Shapiro test were log transformed prior to conducting statistical analysis. Differences in time parameters (*t*_max_) between treatments were assessed by Non-parametric Wilcoxon–Mann–Whitney Rank sum test. The difference between study products in calcium concentration at a respective timepoint was assessed by paired *t*-test. Categorical variables were assessed using Poisson Generalized Linear Model. In the statistical analysis, value of *p* less than 0.05 was considered statistically significant. The data were analyzed using the SAS ® version 9.4 (SAS Institute, Cary, North Carolina).

## 3. Results

### 3.1. Baseline characteristics

**Figure 2:**
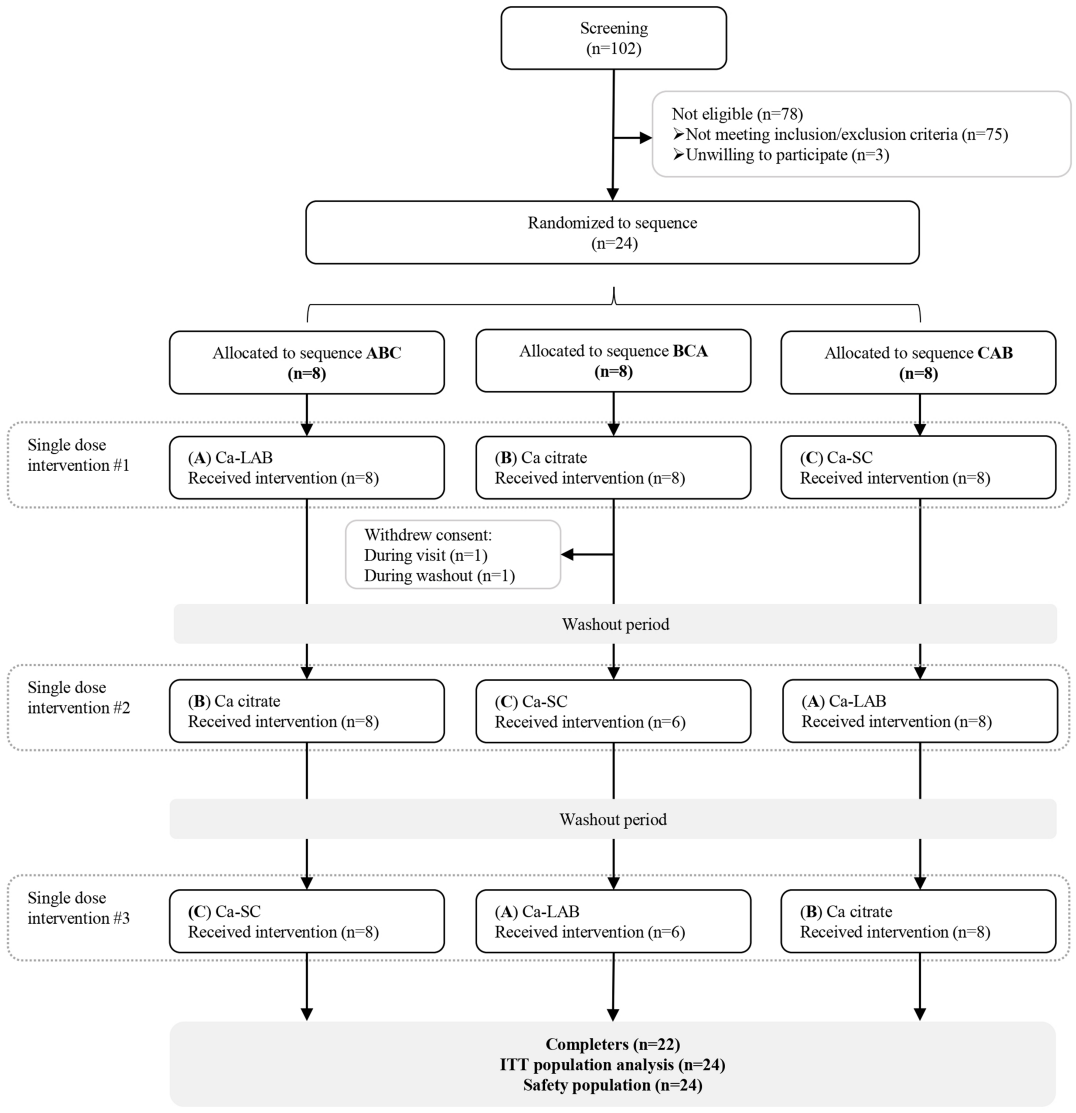
Flow diagram of study participants. Ca = calcium; ITT = Intent-to-treat.

Twenty-four healthy postmenopausal females who met the inclusion and exclusion criteria described in the study protocol were enrolled in the clinical study. A total of 22 completed the study, with two participants withdrawing consent following the first intervention (Figure 2). At baseline, the mean age of the women was 56.2 years, 23 were of European descent and one of southeast Asian descent. Participants were normal weight, with a mean BMI of 24 kg/m^2^ (Table 1).

**Table 1:** Demographic and baseline characteristics of the study participants.

	Total (*n* = 24)	Allocated Sequences		
		A➔B➔C (*n* = 8)	B➔C➔A (*n* = 8)	C➔A➔B (*n* = 8)
	Mean (SD)			
Age (years)	56.2 (4.5)	55.9 (4.7)	57.3 (5.1)	55.4 (3.9)
Years after menopause	6.6 (4.1)	4.8 (3.4)	9.2 (5.1)	5.8 (2.6)
Ethnicity, *n* (%)				
White (Eastern European)	3 (12.5)	2 (25.0)	0 (0.0)	1 (12.5)
White (Western European)	20 (83.3)	6 (75.0)	8 (100)	6 (75.0)
Southeast Asian	1 (4.2)	0 (0.0)	0 (0.0)	1 (12.5)
Weight (kg)	62.0 (6.2)	62.0 (4.7)	62.0 (5.2)	62.1 (8.7)
BMI (kg/m^2^)	24.0 (2.1)	23.7 (2.3)	24.2 (2.3)	24.0 (2.0)
25(OH)D (mmol/L)	91.6 (22.7)	90.1 (23.9)	95.4 (21.6)	89.4 (25.1)
PTH (pmol/L)	4.2 (1.1)	4.6 (0.9)	3.8 (0.9)	4.2 (1.4)

A = calcium-carrying *Lactobacillus* (Ca-LAB); B = calcium citrate; C = calcium-carrying *Saccharomyces cerevisiae* (Ca-SC).

Data are expressed as mean (SD) unless otherwise noted.

### 3.2. Serum calcium

**Figure 3:**
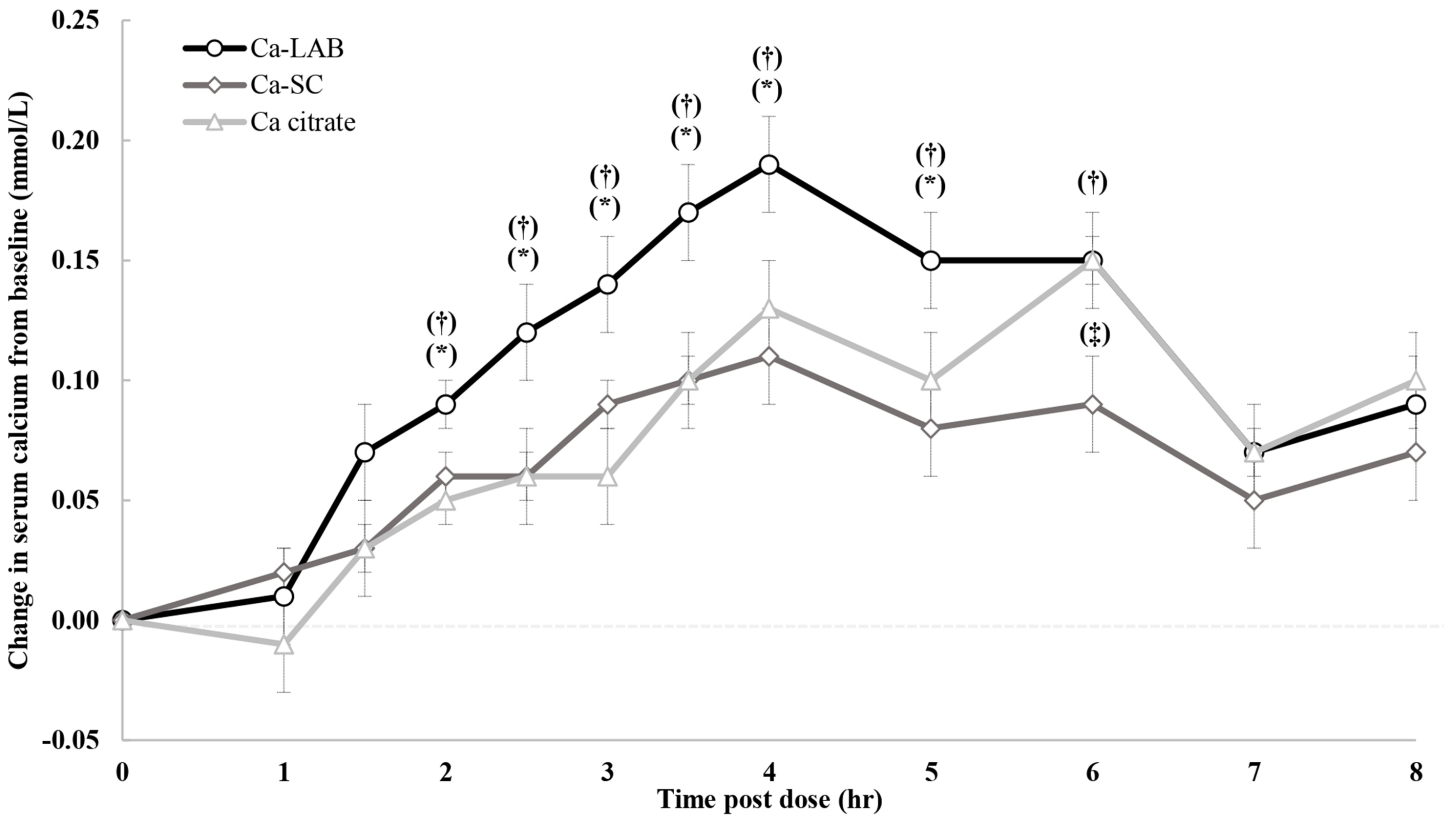
Change in serum calcium concentration from baseline after single oral load of calcium-carrying *Lactobacillus* (Ca-LAB), calcium citrate and *Saccharomyces cerevisiae* (Ca-SC). Vertical bars indicate standard error of the mean. Significant difference between Ca-LAB and calcium citrate or Ca-SC is shown by ^*^p ≤ 0.05 and ^†^*p* ≤ 0.05, respectively. Significant difference between Ca-SC and calcium citrate is shown by ^‡^*p* ≤ 0.05. *p*-value is based on paired *t*-test. Ca = calcium.

The change in serum calcium concentration from pre-intervention over 8 h after administration is shown in Figure 3. Increment in serum calcium concentration was significantly higher following Ca-LAB administration compared to calcium citrate and Ca-SC, from 2 to 5 h post dose and from 2 to 6 h post dose, respectively. Following calcium citrate administration, there was no significant difference in serum calcium concentration compared to Ca-SC post dose up to 5 h but increment serum calcium was significantly higher at 6 h post dose. Comparison of serum calcium pharmacokinetic parameters among the three products are presented in Table 2. Ca-LAB had higher iAUC_0-8h_ compared to calcium citrate (*p* = 0.006) and Ca-SC (*p* = 0.004). For the other pharmacokinetic parameters, the AUC_T0-8h_ of Ca-LAB, was significantly higher than that of Ca-SC (*p* = 0.001) and showed trend toward significance compared to calcium citrate (*p* = 0.060). *C*_max_ was higher for Ca-LAB compared to Ca-SC (*p* < 0.001). *t*_max_ was not significantly different between the three products. There was no difference in pharmacokinetic parameters between calcium citrate and Ca-SC except for *C*_max_ that was substantially higher for calcium citrate (*p* = 0.028).

**Table 2:** Comparison of pharmacokinetic parameters in serum and urine between calcium-carrying *Lactobacillus* (Ca-LAB), calcium citrate, and calcium-carrying *Saccharomyces cerevisiae* (Ca-SC).

	Ca-LAB	Ca-SC	Ca citrate
	Mean (SD)		
Serum			
Pre-dose serum calcium (mmol/L)	2.36 (0.09)	2.35 (0.08)	2.37 (0.08)
iAUC_0-8h_(mmol/L*h)	0.86 (0.51)^a^	0.52 (0.45)^b^	0.60 (0.49)^b^
AUC_T0-8h_ (mmol/L*h)	19.72 (0.74)^a^	19.34 (0.68)^b^	19.14 (2.13)^a,b^
*C*_max_ (mmol/L)	2.57 (0.11)^a^	2.50 (0.10)^b^	2.54 (0.10)^a^
*t*_max_ (h)^†^	4.0 (2.0–7.0)	4.0 (1.5–8.0)	5.5 (2.0–8.0)
Urine			
Pre-dose urine calcium normalized to creatinine (mmol/mmol)	0.29 (0.19)	0.29 (0.18)	0.30 (0.21)
Pre-dose excreted calcium (mg)	5.83 (3.81)	7.27 (13.67)	5.40 (5.30)
AUC_T0-10h_ (mmol/mmol*h)	9.32 (2.91)^a^	7.96 (2.65)^b^	7.50 (2.61)^b^
AUC_T0-24h_ (mmol/mmol*h)	14.08 (4.76)^a^	12.40 (4.36)^b^	11.65 (4.47)^b^
*C*_max_ (mmol/mmol)	1.48 (0.48)^a^	1.22 (0.39)^b^	1.18 (0.33)^b^
*t*_max_ (h)^†^	5.0 (3.0–7.0)	5.0 (3.0–7.0)	5.0 (3.0–9.0)

iAUC, incremental area under the concentration-time curve; Ca, calcium; AUC_T_, area under the concentration-time curve; *C*_max_, peak concentration; *t*_max_, time to peak concentration; h, hours. ^†^*t*_max_ is presented as median (range). ^a,b^Rows values not sharing the same superscript are significantly different from each other, *P* ≤ 0.05. Rows without any superscripts are not significantly different from each other *p* > 0.05.

Data are expressed as mean (SD) unless otherwise noted.

### 3.3. Urine calcium

**Figure 4:**
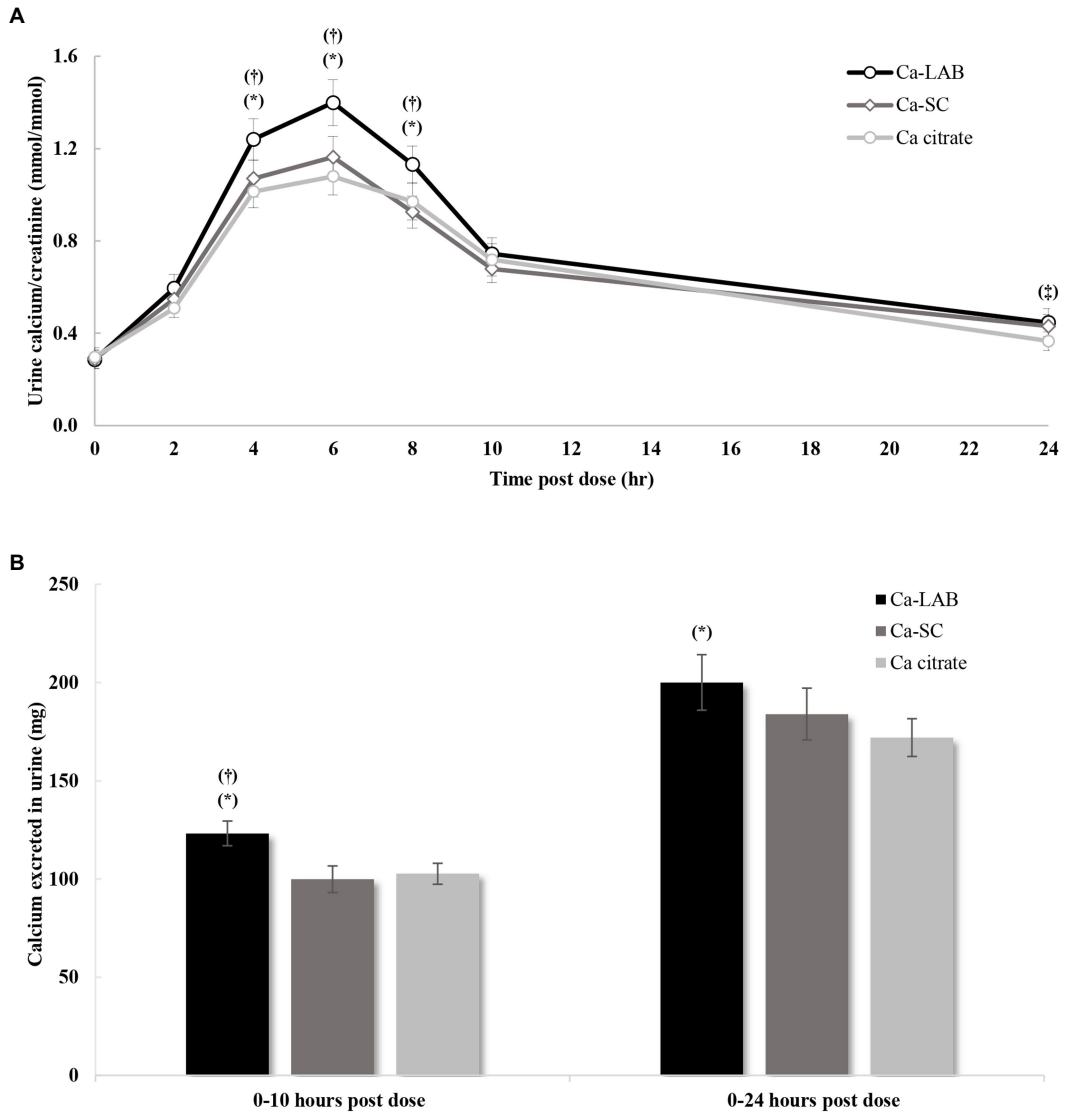
Urinary calcium profile after single oral load of calcium-carrying *Lactobacillus* (Ca-LAB), calcium citrate and calcium-carrying *Saccharomyces cerevisiae* (Ca-SC). **(A)** Urine calcium concentration corrected by corresponding urinary creatinine (mmol/mmol) from 0 to 24 h post dose. **(B)** Total calcium excreted in urine for 10 and 24 h post dose. Vertical bars indicate standard error of the mean. Significant difference between Ca-LAB and calcium citrate or Ca-SC is shown by ^*^p ≤ 0.05 and ^†^*p* ≤ 0.05, respectively. Significant difference between Ca-SC and calcium citrate is shown by ^‡^*p* ≤ 0.05. *p*-value is derived based on paired *t*-test. Ca = calcium.

The change in urinary calcium over the 24-h collection period for the three test products is represented in Figure 4A. Urinary calcium significantly increased following Ca-LAB administration compared to calcium citrate and Ca-SC administrations for urine collected in intervals 2–, 4–, and 6–8 h. Urinary calcium profiles were similar following calcium citrate and Ca-SC administration, with only one significantly higher calcium value for calcium citrate compared to Ca-SC in interval 10–24 h. Total calcium excreted up to 10 h post dose was higher following Ca-LAB administration compared to calcium citrate (*p* = 0.002) and Ca-SC (*p* = 0.004; Figure 4B). Furthermore, urine calcium excretion over 24 h was higher following Ca-LAB administration compared to calcium citrate administration (*p* = 0.013; Figure 4B). Urine calcium pharmacokinetic parameters between the three products are presented in Table 2. AUC_T0-10h_, AUC_T0-24h_, and *C*_max_ were significantly higher for Ca-LAB compared to calcium citrate (*p* < 0.001, *p* = 0.006, and *p* < 0.001, respectively) and Ca-SC (*p* < 0.001, *p* = 0.003, and *p* < 0.001, respectively). No differences were observed between calcium citrate and Ca-SC.

### 3.4. Safety evaluation

A total of 26 AEs was reported in 11 participants throughout the study, four participants following Ca-LAB administration, seven participants following calcium citrate administration, and six participants following Ca-SC administration (Supplementary Table S3). There was neither a statistically significant difference in the proportion of participants reporting AEs, nor in the rate of AE incidences between the study products (data not shown). Out of the 26 AEs, only four gastrointestinal related AEs reported by one participant following the Ca-LAB administration classified as possibly product related (data not shown). The majority of the AEs (73.1%) were mild, and the rest were moderate (Supplementary Table S3). Headache was the most common AE, reported eight times during the study by six participants. No clinically significant changes in clinical chemistry or hematology, anthropometrics or vital signs were observed (data not shown). Both Ca-LAB and Ca-SC were well tolerated by the study participants.

## 4. Discussion

Calcium is an important micronutrient and although it can be absorbed from a varied diet, especially from dairy and certain vegetables, there often may be a need to supplement the diet (14). The biological availability of calcium from dietary sources is variable and may be equivalent or lower than from supplements (16, 17) which highlights there may be a need for alternative dietary supplement solutions. The aim of this study was to compare the bioavailability of a yeast (Ca-SC) and a *Lactobacillus* (Ca-LAB) calcium supplement to a commercially available calcium citrate supplement in a single serving, using a pharmacokinetic approach. The present findings confirmed that calcium in an enriched *Lactobacillus*-based postbiotic system is associated with higher levels of bioavailability as compared to calcium citrate, a conventional salt-based calcium supplement, while calcium in an enriched yeast-based postbiotic system had equivalent bioavailability as calcium citrate. The bioavailability of calcium in serum during the Ca-LAB period was higher than that of calcium citrate and Ca-SC. This was most clearly demonstrated by significantly higher iAUC_0-8h_ for Ca-LAB in comparison to calcium citrate and Ca-SC. This parameter is considered more appropriate for serum measurements compared to total AUC, as the varying baseline values of individuals are taken into consideration (39, 42). The pharmacokinetic profile and parameters of calcium in serum was similar for Ca-SC and calcium citrate except for a significant difference in *C*_max_.

Calcium excretion in urine was also increased with Ca-LAB in comparison to calcium citrate and Ca-SC. This was illustrated by its higher calcium AUC_T0-10h_, AUC_T0-24h_, and *C*_max_, and the total calcium mass excreted at 0–10 h and at 0–24 h while Ca-SC and calcium citrate demonstrated similar urinary excretion. This too indicates a higher bioavailability, as it was previously suggested that oral calcium load is correlated with calcium excretion in urine (43).

Calcium in an enriched cell system is assumed to be naturally integrated by the growing microorganism into its own structure as part of the fermentation process (30, 31, 44). Consequently, the elemental calcium may change its chemical properties and interactions within the matrix, resulting in calcium complexes that are highly bioavailable (16, 29). This is evident by large variation in calcium absorption observed in different type of foods, ranging from 5% in spinach to approximately 50% in *Brassica* vegetables when fed at comparable calcium loads (16, 27). In comparison, calcium carbonate and calcium citrate, the most widely used salt based calcium supplements by consumers, were reported to have similar absorbance around 24% (25). Based on the study results, enriched postbiotic system could potentially promote the buildup of calcium in tissues, however, long-term exposure studies are needed to clarify if more of the absorbed calcium is retained in the body and what type of calcium complexes promote this effect.

Calcium bioavailability, besides being influenced by delivery system, is also controlled by other factors like age, physiological conditions, hormonal regulation and dietary patterns (16, 45). To ensure a reasonably homogeneous group, the target population for this study was healthy postmenopausal women. This population was chosen as one who most likely to be needing an effective calcium supplementation to maintain bone health (9–11, 46). Moreover, participants with vitamin D3 deficiency were excluded from the study, and vitamin D3 content was standardized across all study products. Calcium and sodium intakes were standardized prior to baseline, as both are known for their dietary effects on calcium absorption and excretion (39, 42).

The safety evaluation during the study demonstrated that both Ca-LAB and Ca-SC were well tolerated with no significant difference in adverse events between study products. Majority of the AEs reported in this study (73.1%) were mild with no serious AEs reported. The most common AE was headache, which was observed in six participants after administration of all tested products. Only one participant who received Ca-LAB experienced gastrointestinal AEs that were considered possibly product related. All AEs have been reported in previous bioavailability studies evaluating calcium supplements (47). There were no clinically significant changes in laboratory parameters (clinical chemistry and hematology) or vital signs after intervention of all tested products.

Despite the strengths of this randomized, double-blind, comparator-controlled, crossover study, there are a few inherent limitations. Fractional intestinal calcium absorption, a more precise and accurate method, could not be used since commercial dietary products could not be labeled with calcium isotopes. Moreover, the increment in serum calcium from baseline at 8 h after calcium loads was still substantial for all test groups, indicating that calcium absorption was not completed at 8 h post dose. Thus, elimination rate constant and elimination rate, as well as extrapolation of AUC_T_ to infinity were impractical to measure. Although considered as limitations, this pharmacokinetic study is adequate in assessing relative bioavailability of calcium preparations and is in line with other studies (39, 42, 48).

Lastly, calcium was administered together with a breakfast, therefore, the interaction between calcium preparations and food could not be evaluated as a factor influencing the pharmacokinetic endpoints (49, 50).

To conclude, the present study shows that Ca-LAB had greater calcium bioavailability as compared to calcium citrate and Ca-SC whereas the calcium bioavailability of calcium citrate and Ca-SC was similar. Both Ca-LAB and Ca-SC were well tolerated with no unexpected safety concerns.

## Data availability statement

The datasets presented in this article are not readily available because of the proprietary nature of the dataset, and privacy considerations. Requests to access the datasets should be directed to eran.ivanir@iff.com.

## Ethics statement

The studies involving human participants were reviewed and approved by Advarra Institutional review board (Aurora ON, Canada) and Health Canada’s Natural and Non-prescription Health Products Directorate. The patients/participants provided their written informed consent to participate in this study.

## Author contributions

MF, AH, and EI conceived and designed the study. ME and DC had principal role in performing the protocol. DI, SF-A, and GH analyzed the data. MF, AO, SF-A, GH, and EI wrote the manuscript. All authors contributed to the article and approved the submitted version.

## Funding

This research was funded by International Flavors & Fragrances Inc. (IFF).

## Conflict of interest

This study was fully sponsored by International Flavors & Fragrances Inc. (IFF). IFF designed the study and interpreted the results. MF, AO, and EI are employees of IFF. AH was an employee of IFF during the study. DI, SF-A, and GH are employees of Medistat, a company that was hired by IFF to perform the statistical analysis of the study results. ME and DC are employees of KGK Science Inc. a company contracted by IFF to conduct the study. Ca-SC and Ca-LAB are both manufactured by Grow Company, a company owned by IFF.

## Publisher’s note

All claims expressed in this article are solely those of the authors and do not necessarily represent those of their affiliated organizations, or those of the publisher, the editors and the reviewers. Any product that may be evaluated in this article, or claim that may be made by its manufacturer, is not guaranteed or endorsed by the publisher.

## References

[1] Peacock M. Calcium metabolism in health and disease. Clin J Am Soc Nephrol. (2010) 5:S23–30. doi: 10.2215/CJN.05910809 20089499

[2] Uusi-Rasi K Karkkainen MU Lamberg-Allardt CJ. Calcium intake in health maintenance—a systematic review. Food. Nutr Res. (2013) 57:21082. doi: 10.3402/fnr.v57i0.21082 PMC3657072 23687486

[3] Fleet JC. The role of vitamin D in the endocrinology controlling calcium homeostasis. Mol Cell Endocrinol. (2017) 453:36–45. doi: 10.1016/j.mce.2017.04.008, PMID: 28400273 PMC5529228

[4] Veldurthy V Wei R Oz L Dhawan P Jeon YH Christakos S. Vitamin D, calcium homeostasis and aging. Bone Res. (2016) 4:16041. doi: 10.1038/boneres.2016.41, PMID: 27790378 PMC5068478

[5] Available from: https://ods.od.nih.gov/factsheets/Calcium-HealthProfessional/. September 26, 2018 (cited 2019 May 17, 2019).

[6] Baker JF Davis M Alexander R Zemel BS Mostoufi-Moab S Shults J. Associations between body composition and bone density and structure in men and women across the adult age spectrum. Bone. (2013) 53:34–41. doi: 10.1016/j.bone.2012.11.035, PMID: 23238122 PMC3552077

[7] Greendale GA Edelstein S Barrett-Connor E. Endogenous sex steroids and bone mineral density in older women and men: the rancho Bernardo study. J Bone Miner Res. (1997) 12:1833–43. doi: 10.1359/jbmr.1997.12.11.1833, PMID: 9383688

[8] Riggs BL Khosla S Melton LJ 3rd. Sex steroids and the construction and conservation of the adult skeleton. Endocr Rev. (2002) 23:279–302. doi: 10.1210/edrv.23.3.0465, PMID: 12050121

[9] Albright FSPH Richardson AM. Postmenopausal osteoporosis: it's clinical features. JAMA. (1941) 116:2465–74. doi: 10.1001/jama.1941.02820220007002

[10] Cauley JA. Estrogen and bone health in men and women. Steroids. (2015) 99:11–5. doi: 10.1016/j.steroids.2014.12.010 25555470

[11] Lindsay R. Letter: Estrogen and bone loss. Arch Intern Med. (1976) 136:1068. doi: 10.1001/archinte.1976.03630090090027 962453

[12] Chen JS Sambrook PN. Antiresorptive therapies for osteoporosis: a clinical overview. Nat Rev Endocrinol. (2011) 8:81–91. doi: 10.1038/nrendo.2011.146, PMID: 21894214

[13] Ross A.C., et al. Dietary reference intakes for calcium and vitamin D. (2011). Available from: https://www.ncbi.nlm.nih.gov/pubmed/21796828. 21796828

[14] Plantz M.A. Bittar K.. Dietary calcium. StatPearls. (2022). Available from: https://www.ncbi.nlm.nih.gov/pubmed/31747199. 31747199

[15] Bailey RL Dodd KW Goldman JA Gahche JJ Dwyer JT Moshfegh AJ. Estimation of total usual calcium and vitamin D intakes in the United States. J Nutr. (2010) 140:817–22. doi: 10.3945/jn.109.118539, PMID: 20181782 PMC2838624

[16] Shkembi B Huppertz T. Calcium absorption from food products: food matrix effects. Nutrients. (2021) 14:180. doi: 10.3390/nu14010180, PMID: 35011055 PMC8746734

[17] Wolf RL Cauley JA Baker CE Ferrell RE Charron M Caggiula AW. Factors associated with calcium absorption efficiency in pre-and perimenopausal women. Am J Clin Nutr. (2000) 72:466–71. doi: 10.1093/ajcn/72.2.466, PMID: 10919942

[18] Jackson RD LaCroix AZ Gass M Wallace RB Robbins J Lewis CE. Calcium plus vitamin D supplementation and the risk of fractures. N Engl J Med. (2006) 354:669–83. doi: 10.1056/NEJMoa055218 16481635

[19] Curhan GC Willett WC Speizer FE Spiegelman D Stampfer MJ. Comparison of dietary calcium with supplemental calcium and other nutrients as factors affecting the risk for kidney stones in women. Ann Intern Med. (1997) 126:497–504. doi: 10.7326/0003-4819-126-7-199704010-00001, PMID: 9092314

[20] Lewis JR Zhu K Prince RL. Adverse events from calcium supplementation: relationship to errors in myocardial infarction self-reporting in randomized controlled trials of calcium supplementation. J Bone Miner Res. (2012) 27:719–22. doi: 10.1002/jbmr.1484, PMID: 22139587

[21] Heaney RP Recker RR Stegman MR Moy AJ. Calcium absorption in women: relationships to calcium intake, estrogen status, and age. J Bone Miner Res. (1989) 4:469–75. doi: 10.1002/jbmr.5650040404, PMID: 2816496

[22] Scopacasa F Wishart JM Horowitz M Morris HA Need AG. Relation between calcium absorption and serum calcitriol in normal men: evidence for age-related intestinal resistance to calcitriol. Eur J Clin Nutr. (2004) 58:264–9. doi: 10.1038/sj.ejcn.1601777, PMID: 14749746

[23] Melse-Boonstra A. Bioavailability of micronutrients from nutrient-dense whole foods: zooming in on dairy, vegetables, and fruits. Front Nutr. (2020) 7:101. doi: 10.3389/fnut.2020.00101, PMID: 32793622 PMC7393990

[24] Charles P. Calcium absorption and calcium bioavailability. J Intern Med. (1992) 231:161–8. doi: 10.1111/j.1365-2796.1992.tb00519.x 1541940

[25] Zhang YY Stockmann R Ng K Ajlouni S. Opportunities for plant-derived enhancers for iron, zinc, and calcium bioavailability: a review. Compr Rev Food Sci Food Saf. (2021) 20:652–85. doi: 10.1111/1541-4337.12669, PMID: 33443794

[26] Rafferty K Walters G Heaney R. Calcium fortificants: overview and strategies for improving calcium nutriture of the US population. J Food Sci. (2007) 72:R152–8. doi: 10.1111/j.1750-3841.2007.00521.x, PMID: 18034744

[27] Weaver CM. Calcium in food fortification strategies. Int Dairy J. (1998) 8:443–9. doi: 10.1016/S0958-6946(98)00067-3

[28] Camara-Martos F Amaro-López M. Influence of dietary factors on calcium bioavailability. Biol Trace Elem Res. (2002) 89:43–52. doi: 10.1385/BTER:89:1:43 12413050

[29] Vinson J. Comparison of different forms of calcium on blood pressure of normotensive young males. Nut Rep Intl. (1987) 36:497–505.

[30] Vinson JA Tompkins TA Agbor GA. Comparative bioavailability of mineral-enriched gluconates and yeast in rat liver after depletion-repletion feeding. Biol Trace Elem Res. (2007) 118:104–10. doi: 10.1007/s12011-007-0004-1, PMID: 17873352

[31] Zhang S Zhang Y Peng N Zhang H Yao J Li Z. Pharmacokinetics and biodistribution of zinc-enriched yeast in rats. Sci World J. (2014) 2014:1–4. doi: 10.1155/2014/217142, PMID: 25215316 PMC4151581

[32] EFSA. Scientific opinion on chromo precise® cellular bound chromium yeast added for nutritional purposes as a source of chromium in food supplements and the bioavailability of chromium from this source. EFSA J. (2012) 10:2951. doi: 10.2903/j.efsa.2012.2951

[33] Moher D Hopewell S Schulz KF Montori V Gøtzsche PC Devereaux PJ. CONSORT 2010 explanation and elaboration: updated guidelines for reporting parallel group randomised trials. J Clin Epidemiol. (2010) 63:e1–e37. doi: 10.1016/j.jclinepi.2010.03.004, PMID: 20346624

[34] Guideline IH. Integrated addendum to ICH E6 (R1): guideline for good clinical practice E6 (R2). Curr Step. (2015) 2:1–60.

[35] Guo L Harnedy PA Li B Hou H Zhang Z Zhao X. Food protein-derived chelating peptides: biofunctional ingredients for dietary mineral bioavailability enhancement. Trends Food Sci Technol. (2014) 37:92–105. doi: 10.1016/j.tifs.2014.02.007

[36] Cormick G Belizán JM. Calcium intake and health. Nutrients. (2019) 11:1606. doi: 10.3390/nu11071606, PMID: 31311164 PMC6683260

[37] Vatanparast H Islam N Patil RP Shafiee M Whiting SJ. Calcium intake from food and supplemental sources decreased in the Canadian population from 2004 to 2015. J Nutr. (2020) 150:833–41. doi: 10.1093/jn/nxz318, PMID: 31891395 PMC7138660

[38] GBD 2017 Diet Collaborators. Health effects of dietary risks in 195 countries, 1990–2017: a systematic analysis forthe global burden of disease study. Lancet. (2019) 393:1958–72. doi: 10.1016/S0140-6736(19)30041-8 30954305 PMC6899507

[39] Heller HJ Greer LG Haynes SD Poindexter JR Pak CYC. Pharmacokinetic and pharmacodynamic comparison of two calcium supplements in postmenopausal women. J Clin Pharmacol. (2000) 40:1237–44. doi: 10.1177/009127000004001108, PMID: 11075309

[40] Grow Nutrients®|IFF Health. Available from: https://iff-health.com/portfolio/grow-nutrients-pp/.

[41] Wang H Bua P Capodice J. A comparative study of calcium absorption following a single serving administration of calcium carbonate powder versus calcium citrate tablets in healthy premenopausal women. Food. Nutr Res. (2014) 58:23229. doi: 10.3402/fnr.v58.23229 PMC3999951 24772062

[42] Heller HJ Stewart A Haynes S Pak CYC. Pharmacokinetics of calcium absorption from two commercial calcium supplements. J Clin Pharmacol. (1999) 39:1151–4. doi: 10.1177/009127009903901106, PMID: 10579145

[43] Nicar MJ Pak CY. Calcium bioavailability from calcium carbonate and calcium citrate. J Clin Endocrinol Metab. (1985) 61:391–3. doi: 10.1210/jcem-61-2-391, PMID: 4008614

[44] Rayman MP. The use of high-selenium yeast to raise selenium status: how does it measure up? Br J Nutr. (2004) 92:557–73. doi: 10.1079/BJN20041251, PMID: 15522125

[45] Pereira GA. Dietary calcium: strategies to optimize intake. Rev Bras Reumatol. (2009) 49:164–71. doi: 10.1590/S0482-50042009000200008

[46] Vaisman N Shaltiel G Daniely M Meiron OE Shechter A Abrams SA. Increased calcium absorption from synthetic stable amorphous calcium carbonate: double-blind randomized crossover clinical trial in postmenopausal women. J Bone Miner Res. (2014) 29:2203–9. doi: 10.1002/jbmr.2255, PMID: 24753014

[47] Wiria M Tran HM Nguyen PHB Valencia O Dutta S Pouteau E. Relative bioavailability and pharmacokinetic comparison of calcium glucoheptonate with calcium carbonate. Pharmacol Res Perspect. (2020) 8:e00589. doi: 10.1002/prp2.589, PMID: 32302064 PMC7164401

[48] Tondapu P Provost D Adams-Huet B Sims T Chang C Sakhaee K. Comparison of the absorption of calcium carbonate and calcium citrate after roux-en-Y gastric bypass. Obes Surg. (2009) 19:1256–61. doi: 10.1007/s11695-009-9850-6, PMID: 19437082 PMC4469176

[49] Heaney RP Recker RR Weaver CM. Absorbability of calcium sources: the limited role of solubility. Calcif Tissue Int. (1990) 46:300–4. doi: 10.1007/BF02563819, PMID: 2110852

[50] Pak CY Avioli LV. Factors affecting absorbability of calcium from calcium salts and food. Calcif Tissue Int. (1988) 43:55–60. doi: 10.1007/BF02555147, PMID: 3142667

