# Supplementary material for: Bioavailability of calcium in an enriched postbiotic system compared to calcium citrate in healthy postmenopausal females; A randomized, double-blind, comparator-controlled, crossover study
Source: Front Nutr. 2023 Mar 15;10:1073622. doi: 10.3389/fnut.2023.1073622 (PMC10050718; doi:10.3389/fnut.2023.1073622)
Supplement: Supplementary file 1 [file Table_1.DOCX]

Supplementary Material

**SUPPLEMENTARY TABLES:**

**Table S1:** Study inclusion and exclusion criteria

**Table S2:** Excluded medications, supplements, and foods/drinks

**Table S3:** Summary of adverse events

**Table S1.** Inclusion and exclusion criteria

| **Inclusion criteria** |
| --- |
| 1. Healthy females between 45 and 65 years of age 2. Absence of menstrual period via natural processes for at least 12 months 3. Body mass index (BMI) between 19.0 to 29.9 kg/m^2^, inclusive 4. Normal electrocardiogram (ECG) as assessed by a qualified investigator (QI) 5. Parathyroid hormone 1-84 level between 1.6 and 6.9 pmol/L 6. 25(OH)D level ≥20 ng/mL 7. Consumes a normal calcium intake of approximately greater than 450 mg of calcium per day from diet and supplementation as assessed by a nutritionist 8. Willingness to complete records and diaries associated with the study and to complete all clinical visits 9. Agrees to consume the standardized meals 10. Agrees to maintain a diet containing less than 400 mg/day of calcium and 2300 mg/day of sodium from 7 days prior to baseline and until the end of study 11. Agrees to maintain their exercise routine and sun exposure throughout the study 12. Provide voluntary, written informed consent to participate in the study 13. Healthy as determined by medical history, laboratory results, and physical exam as assessed by a QI. |
| **Exclusion criteria** |
| 1. Women who are pregnant, breastfeeding or planning to become pregnant during the trial. 2. Allergy, sensitivity, or intolerance to the investigational product’s active, inactive ingredients or the ingredients of the standardized meals. 3. Verbal confirmation of the diagnosis of osteoporosis. 4. Any disease that may affect calcium or vitamin D metabolism: hyper or hypocalcemia; hyper or hypoparathyroidism; hyperthyroidism or hypothyroidism. 5. Hyperlipidemia as defined by LDL >3.36 mmol/L (130 mg/dL) and/or triglycerides >2.26 mmol/L (200 mg/dL). 6. Surgically induced menopause. 7. Subjects that have followed a specific diet (ketogenic, paleo, etc.) or a change in diet in the past 30 days from baseline. 8. Difficulty swallowing capsules. 9. Unstable metabolic disease or chronic diseases as assessed by the QI. 10. Unstable hypertension. Treatment on a stable dose of medication for at least 3 months was considered by the QI. 11. Pre-diabetic (as defined by glycosylated hemoglobin [HbA1c] >5.7%), Type I diabetes, or Type II diabetes. 12. A significant cardiovascular event in the past 6 months. Subjects with a significant cardiovascular event greater than 6 months ago and if on stable medication may be included after assessment by the QI on a case-by-case basis. 13. Major surgery in the past 3 months or individuals who have planned surgery during the course of the trial. Subjects with minor surgery was considered on a case-by-case basis by the QI 14. Cancer, except skin cancers completely excised with no chemotherapy or radiation with a follow up that is negative. Volunteers with cancer in full remission for more than five years after diagnosis are acceptable. 15. Individuals with an autoimmune disease or immune-compromised individuals. 16. Verbal confirmation of a human immunodeficiency virus (HIV), hepatitis B- and/or hepatitis C-positive diagnosis 17. History of or current diagnosis with kidney disease (e.g., renal failure, kidney transplant, nephrolithiasis or dialysis) and/or liver diseases which may impact study outcomes. 18. Verbal confirmation of medical or neuropsychological condition and/or cognitive impairment that, in the QI’s opinion, could interfere with study participation. 19. Current or history of any significant diseases of the gastrointestinal tract (e.g., intestinal malabsorption, inflammatory bowels disease, chronic diarrhea, or celiac disease). 20. Verbal confirmation of current ongoing blood/bleeding disorder. The disorder was assessed by the QI. 21. An acute gout attack within the past 3 months. 22. Current use of prescribed medications listed in **table S2.A** 23. Current use of over-the-counter medications, supplements, foods and/or drinks listed in **table S2.B** 24. Use of medical cannabinoid products. 25. Chronic use of recreational cannabinoid products (>2 times/week). Occasional use to be assessed by QI on a case-by-case basis. 26. Use of tobacco products within 60 days of baseline. 27. Alcohol or drug abuse within the last 12 months from baseline. 28. High alcohol intake (average of >2 standard drinks per day or >10 standard drinks per week). 29. Clinically significant abnormal laboratory results at screening as assessed by the QI 30. Blood donation 30 days prior to screening, during the study, or a planned donation within 30-days of the last study visit. 31. Participation in other clinical research trials 30 days prior to screening. 32. Individuals who are unable to give informed consent. 33. Any other active or unstable medical condition, that, in the opinion of the QI, may adversely affect the subject's ability to complete the study or its measures or pose a significant risk to the subject |

**Table S2.** Excluded medications, supplements, and foods/drinks

| 1. **Prescribed medications** |
| --- |
| 1. Antibiotics (allowed following 30-day washout) 2. Calcium channel blockers 3. Bisphosphonates, Denosumab (Prolia), and other medications taken for osteoporosis 4. Calcipotriene (Dovonex) 5. Digoxin (Lanoxin) 6. Lithium 7. Beta blockers 8. Estrogen, including topical creams 9. Anticholesteremic (lipid-lowering) medications (e.g. statins, bile acid sequestrants) 10. Diuretics 11. Glucocorticoids 12. Proton Pump Inhibitors 13. Fluoride 14. Diuretics 15. Laxatives 16. Any other medication known as affecting calcium metabolism |
| 1. **Over-the-counter medications, supplements, and foods/drinks** |
| 1. Calcium-containing supplements (allowed following 2 weeks washout) 2. Vitamin D-containing supplements (allowed following 4 weeks washout) 3. Multivitamins (allowed following 2 weeks washout) 4. Mineral supplements (allowed following 2 weeks washout) 5. Calcium and/or aluminum-containing antacids (allowed following 2 weeks washout) 6. Red yeast rice (allowed following 4 weeks washout) 7. Phytosterol and isoflavone supplements (allowed following 3 weeks washout) 8. Diuretics (allowed following 2 weeks washout) 9. Laxatives (allowed following 2 weeks washout) 10. Vitamin D-fortified dairy products (allowed following 2 weeks washout) 11. Calcium-fortified orange juice and dairy products (allowed following 2 weeks washout) 12. Gruyere, Swiss, goat, low-fat cheddar, and mozzarella cheese (allowed following 7 days washout) 13. Dark green leafy vegetables (collards, spinach, kale, turnip greens (allowed following 7 days washout) 14. Canned fish with bones (allowed following 7 days washout) 15. Tofu (allowed following 7 days washout) 16. Sesame seed paste (allowed following 7 days washout) |

**Table S3.** Summary of adverse events based on severity and causality

|  | **Ca-LAB** | | **Ca-SC** | | **Ca citrate** | | **All subjects** | |
| --- | --- | --- | --- | --- | --- | --- | --- | --- |
|  | (n = 24) | | (n = 24) | | (n = 24) | | (n = 24) | |
|  | Subjects  N (%) | Events  N (%) | Subjects  N (%) | Events  N (%) | Subjects  N (%) | Events  N (%) | Subjects  N (%) | Events  N (%) |
| **Total AEs** | 4 (16.7) | 11 (100) | 6 (25) | 7 (100) | 7 (29.2) | 8 (100) | 11 (45.8) | 26 (100) |
| **Mild AEs** | 4 (16.7) | 11 (100) | 3 (12.5) | 4 (57.1) | 3 (12.5) | 4 (50) | 7 (29.2) | 19 (73.1) |
| **Moderate AEs** | 0 | 0 | 3 (12.5) | 3 (42.9) | 4 (16.7) | 4 (50) | 6 (25.0) | 7 (26.9) |
| **Possibly product-related AEs** | 1 (4.2) | 4 (36.4) | 0 | 0 | 0 | 0 | 1 (4.2) | 4 (15.4) |
